# A predictive model to distinguish malignant and benign thyroid nodules based on age, gender and ultrasonographic features^☆^

**DOI:** 10.1016/j.bjorl.2017.10.001

**Published:** 2017-11-04

**Authors:** Fábio Muradás Girardi, Laura Mezzomo da Silva, Cecilia Dias Flores

**Affiliations:** a Complexo Hospitalar Santa Casa, Hospital Santa Rita, Departamento de Cirurgia de Cabeça e Pescoço, Porto Alegre, RS, Brazil; b Complexo Hospitalar Santa Casa, Departamento de Radiologia, Porto Alegre, RS, Brazil; c Universidade Federal de Ciências da Saúde de Porto Alegre (UFCSPA), Departamento de Informática, Porto Alegre, RS, Brazil

**Keywords:** Thyroid nodule, Thyroid neoplasms, Ultrasonography, Cytology, Biopsy needle, Nódulo da tireoide, Neoplasias da tireoide, Ultrassonografia, Citologia, Biópsia por agulha

## Abstract

**Introduction:**

A discussion in literature about a standardized decision support tool for the management of thyroid nodules remains.

**Objective:**

The purpose of this study was to create a statistical prediction model for thyroid nodules management.

**Methods:**

Two hundred and four benign and 57 malignant thyroid nodules were selected for a retrospective study. The variables age, gender and ultrasonographic features were examined using univariate and multivariate models. A statistical formula was used to calculate the risk of cancer of each case.

**Results:**

In multivariate analysis, irregular shape, absence of halo, lower mean age, homogeneous echotexture, microcalcifications and solid content were associated with cancer. After applying the formula, 20 cases (7.6%) with a calculated risk for malignancy ≤3.0% were found, all of them benign. Setting the calculated risk in ≥80%, 21 (8.0%) cases were selected, and in 85.7% of them cancer was confirmed in histopathology. Internal accuracy of the prediction formula was 92.5%.

**Conclusions:**

The prediction formula reached high accuracy and may be an alternative to other decision support tools for thyroid nodule management.

## Resumo

**Introdução:**

Persiste na literatura uma discussão sobre uma ferramenta padronizada de apoio à decisão para o manejo de nódulos tireoidianos.

**Objetivo:**

Criar um modelo de previsão estatística para o manejo de nódulos tireoidianos.

**Método:**

Foram selecionados 204 casos de nódulos tireoidianos benignos e 57 malignos para o estudo retrospectivo. As variáveis idade, sexo e características ultrassonográficas foram analisadas com modelos univariados e multivariados. Uma fórmula estatística foi usada para calcular o risco de câncer de cada caso.

**Resultados:**

Na análise multivariada, a forma irregular, a ausência de halo, menor idade média, ecotextura homogênea, microcalcificações e conteúdo sólido foram associadas ao câncer. Após a aplicação da fórmula, foram encontrados 20 casos (7,6%) com risco calculado de malignidade ≤ 3,0%, todos benignos. Definiu-se o risco calculado em ≥ 80%, 21 casos (8,0%) foram selecionados e em 85,7% deles o câncer foi confirmado pela histopatologia. A precisão interna da fórmula de previsão foi de 92,5%.

**Conclusões:**

A fórmula de previsão alcançou alta precisão e pode ser uma opção para outras ferramentas de apoio à decisão para o manejo de nódulos da tireoide.

## Introduction

The incidence of thyroid cancer has been rising around the world.^1^, ^2^, ^3^ Despite the high prevalence of thyroid nodules (19–67% on ultrasonography – US), most of them are benign. Only about 5–10% of diagnosed nodules are malignant, although it is well known that this frequency may be higher when considering occasional diagnosis of small microcarcinomas.^4^, ^5^, ^6^

The investigation of these lesions usually requires clinical and imaging examination of the neck, sometimes associated with fine-needle aspiration biopsy (FNAB). Among these imaging tests, US are a safe, cheap, noninvasive and non-radioactivity tool, able to detect and qualitatively evaluate the nodules. To this date, no US sign showed to be pathognomonic of malignancy, however, the combination of several characteristics may help determinate the malignancy risk of a nodule.^7^, ^8^, ^9^

Different US sensitivities, specificities, negative and positive predictive values have been observed. There are variations in US terminology and malignancy criteria as well as an overlap between the US features of malignant and benign nodules among the different studies. Clinical features are not commonly used when applying prediction models.^10^, ^11^ Moreover, verification bias frequently occurs, as many studies are not designed so that all FNAB diagnoses are verified by surgery or clinical observation.

Some well-designed studies investigated the reliability of US findings in comparison with histopathology.^7^, ^10^, ^12^, ^13^, ^14^ Three studies used a formula based on the analysis of US features to predict malignancy.^7^, ^15^, ^16^ Nevertheless, none of them included clinical characteristics. Park et al. proposed a predictive model based on a logit formula, stratifying each lesion into different approach categories, allowing it to be used in future decision analyses.^16^ A model similar to the one used by Park et al. was applied in a sample of surgically treated cases in the authors’ service, developing a statistical decision support tool, based on gender, age and US features. Internal analysis according to pre-operatory cytology was also performed.

## Methods

All patients who had undergone thyroidectomy between January 2009 and December 2013, whose US and USG-FNAB had been performed in the authors’ institution were retrospectively evaluated. Institutional review board approval was obtained (3593/11).

During the studied period, 192 patients were included, corresponding to 261 nodules. Each nodule analyzed as an individual case. Histopathological examination was performed by the same professional (MBB) in 238 (91.1%) cases. In 86 cases (32.9%) surgery was indicated because of goiter with compressive symptoms or relative indications (as large nodules in younger patients); in 67 cases (25.6%), because of nodules with undetermined cytology; in 47 cases (18.0%), because of solid nodules with repeated non-diagnostic cytology; and in 61 cases (23.3%), because of nodules cytologically (45–17.2%) or clinically suspicious for cancer (16–6.1%).

B-mode US and color Doppler examinations were performed, using Toshiba equipment model Xario (SSA 660A), with a high-resolution linear transducer (7.5–14 MHz). FNAB was performed with US guiding, using a 24 gauge needle. When in the presence of a multi-nodular goiter, samples were collected from nodules with the highest index of suspicion on ultrasonography. In partially cystic nodules, the needle was directed into the solid portion. As the structure of this study was modeled in 2008, the utilization of the old Bethesda rating was chosen. The cytological diagnoses were classified in: (I) non-diagnostic; (II) benign; (III) undetermined; (IV) suspicious for malignancy; and (V) malignant. In cases with more than two cytological results in one patient, the result most likely to be malignant was assigned. After histopathological study, resected nodules were classified as: (I) malignant (papillary carcinoma, follicular carcinoma, anaplastic carcinoma, poorly differentiated and medullary carcinoma); or (II) benign (nodular hyperplasia, colloidal goiter, nodular lymphocytic or Hashimoto's thyroiditis, and follicular adenoma).

US findings, gender and age of all patients were recovered from files. All cytological and histological results from patients submitted to thyroidectomies were recovered. The following variables were inserted into a specific database: age, gender, US findings, cytological and histological results. US features of nodules were classified for: (I) echogenicity (marked hypoechoic, predominantly hypoechoic, predominantly isoechoic, predominantly hyperechoic, or predominantly anechoic); (II) internal content (predominantly solid (liquid portion ≤10% of the nodule volume); mixed solid-cystic (liquid portion >10% but ≤50% of the nodule volume); predominantly cystic (liquid portion >50%, but ≤90% of the nodule volume); purely cystic (liquid portion >90% of the nodule volume)); (III) echotexture (homogeneous or heterogeneous); (IV) calcification (microcalcifications, macrocalcifications or peripheral rim calcifications, also called ‘eggshell’ calcifications); (V) halo (present and complete; partially present; or absent); (VI) margins (“defined” or “undefined”); (VII) shape (regular, irregular or lobulated); (VIII) vascular flow (predominantly central; predominantly peripheral; mixed central and peripheral; or absent); (IX) location of the nodule or affected lobe (right lobe, isthmus, or left lobe).

Frequencies and distribution of each selected variable were calculated. The authors used mean (Standard Deviation – SD), absolute frequencies and percentages, as appropriate. For differences between groups, the authors used Chi-square tests for categorical variables and Student's *t*-test for continuous variables. Logistic regression was used to identify US characteristics independently associated with malignancy (dependent variable). The level of statistical significance was set at 5%. All statistical analyses were performed by the software SPSS, version 15.0 (SPSS Inc., Chicago, IL).

A formula was used to calculate the probability of cancer based on the multiple regression analysis results: Probability(*Z*) = 1/1 + *e*^−(*α*+∑*βiXi*)^; where “*e*” and “*α*” represent mathematical constants; and “*β*”, the coefficient of each independent variable (“*X*”).

Applying the statistical tool, the authors could observe a varying risk of malignancy depending on the variables setting. The mathematical formula for risk prediction was applied in all analyzed cases, which were stratified into low risk, intermediate risk, and high risk of malignancy, assuming specific cut-points adapted to the obtained results. Internal analyses according to cytological results were made.

## Results

Sample US features are summarized in Table 1. The patients mean age was 50.06 years (ranging from 13 to 87 years), with a male-to-female ratio of 1:7.7. The mean nodule size was 2.17 cm (ranging from 0.3 to 6.6 cm).

**Table 1:** Univariate analysis: clinical and ultrasonographic features among malignant and benign cases.

	Benign		Malignant		Total		*p*-value
	Mean (SD)		Mean (SD)		Mean (SD)		
*Age*	51.04 (13.41)		46.54 (15.00)		50.06 (13.87)		0.031
*Diameter*	2.31 (1.25)		1.68 (1.14)		2.17 (1.25)		0.004
	*n* = 204	% = 78.1	*n* = 57	% = 21.8	*n* = 261	% = 100	
*Gender (M/F)*	23/181	11.2/88.7	7/50	12.2/87.7	30/231	11.4/88.5	0.867
*Position*							0.125
Left lobe	94	46.0	21	36.8	115	44.0	
Right lobe	79	38.7	21	36.8	100	38.3	
Isthmus	31	15.1	14	24.5	45	17.2	
NI	0	0	1	1.7	1	0.3	
*Content*							<0.001
Solid	108	52.9	47	82.4	155	59.3	
Mixed pred. cystic	2	0.9	0	0	2	0.7	
Mixed pred. solid	73	35.7	7	12.2	80	30.6	
Cystic	21	10.2	3	5.2	24	9.1	
*Echotexture*							0.403
Heterogeneous	36	17.6	7	12.2	43	16.4	
Homogeneous	168	82.3	50	87.7	218	83.5	
*Echogenicity*							<0.001
Pred. anechoic	22	10.7	2	3.5	24	9.1	
Pred. hypoechoic	47	23.0	7	12.2	54	20.6	
Pred. hyperecoic	11	5.3	0	0	11	4.2	
Pred. isoechoic	85	41.6	11	19.2	96	36.7	
Markedly hypoechoic	36	17.6	36	63.1	72	27.5	
NI	3	1.4	1	1.7	4	1.5	
*Halo*							<0.001
Absent	55	26.9	35	61.4	90	34.4	
Regular	106	51.9	18	31.5	124	47.5	
Irregular	43	21.0	4	7.0	47	18.0	
*Margins*							0.001
Well defined	197	96.5	51	89.4	248	95.0	
Ill defined	7	3.4	6	10.5	13	4.9	
*Shape*							<0.001
Regular	178	87.2	29	50.8	207	79.3	
Irregular	12	5.8	17	29.8	29	11.1	
Lobulated	14	6.8	11	19.2	25	9.5	
*Calcifications*							<0.001
Absent	160	78.4	22	38.5	182	69.7	
Macrocalcifications	11	5.3	5	8.7	16	6.1	
Peripheral (“eggshell”)	9	4.4	1	1.7	10	3.8	
Microcalcifications	24	11.7	29	50.8	53	20.3	
*Vascular flow*							0.959
Absent	10	4.9	3	5.2	13	4.9	
Intranodular	102	50.0	25	43.8	127	48.6	
Perinodular	59	28.9	17	29.8	76	29.1	
Peri-intranodular	23	11.2	6	10.5	29	11.1	
NI	10	4.9	6	10.5	16	6.1	

*n*, absolute frequency; %, relative frequency; SD, Standard Deviation; age in years; diameter in centimeters; *p*-value, level of significance used; NI, not informed; M/F, male/female; Pred, predominant.

Malignancy was found after histopathological study in 57 (21.8%) resected nodules (55 papillary carcinoma and 2 follicular carcinoma). Multifocality was found in 24 (9.1%) cases. Among benign diagnoses, 45 (22.0%) were follicular adenoma; 132 (64.7%), follicular hyperplasia; 10 (4.9%), colloid nodules; and 17 (8.3%), nodular form of Hashimoto's thyroiditis. FNAB results were benign in 95 (36.3%) nodules; suspicious, in 16 (6.1%); malignant, in 30 (11.4%); undetermined, in 73 (27.9%); and non-diagnostic, in 47 (18.0%).

Based on the histopathological and ultrasonography description, it was possible to determine, in cases of multinodular goiters, the histology of each nodule submitted to FNAB. Among all benign confirmed cases on histopathology, an occasionally diagnosed papillary thyroid carcinoma was found in other parts of the gland in 26 (9.9%) cases (nodules that were not the subject of the investigation). The mean diameter of occasionally diagnosed carcinomas was 0.73 cm (variation of 0.2–2.4 cm). Among them, 23 (95.8%) were microcarcinomas.

In univariate analysis, the following features were associated to malignancy: lower mean age (*p* = 0.031), lower diameter (*p* = 0.004), solid content (*p* < 0.001), absence of halo (*p* < 0.001), irregular or lobulated shape (*p* < 0.001 and *p* < 0.041, respectively), microcalcification (*p* < 0.001), hypoechoic texture (*p* < 0.001), and ill-defined margins (*p* = 0.001) (Table 1). In multivariate analysis, irregular shape (*p* = 0.039), absence of halo (*p* = 0.016), lower mean age (*p* = 0.020), homogeneous echotexture (*p* = 0.019), microcalcification (*p* = 0.014), and solid content (*p* = 0.007) were associated with cancer (Table 2). With the regression analysis results, the authors elaborated an equation to calculate the risk of cancer of a determined thyroid nodule (*z*), as follows below. The authors found inconsistence's when worked with the variable echogenicity in multivariate analysis. The authors also considered diameter as a selection bias. Both variables were excluded from the equation:

$$Z=\frac{1}{1+\exp(-(-4.642+0.465*X1–0.033*X2+0.916*X3+0.353*X4–0.061*X5+1.475*X6+1.600*X7+1.708*X8+0.889*X9–0.283*X10+1.929*X11+0.762*X12+0.418*X13+1.461*X14+2.133*X15–0.898*X16–0.817*X17–0.078*X18))}*100$$

**Table 2:** Independent clinical and ultrasonographic factors associated to malignity after multiple regressions.

	*β*	SE	*p*-value	OR	95% CI	
					Lower	Upper
Irregular shape	0.762	0.629	0.016	6.884	1.434	33.044
Microcalcification	2.066	1.222	0.014	7.895	0.719	86.660
Absent halo	1.929	0.800	0.016	5.522	1.370	22.249
Homogeneous echotexture	1.601	0.682	0.019	4.956	1.302	18.858
Lower mean age	0.034	0.015	0.020	0.967	0.940	0.995
Solid content	1.475	0.551	0.007	4.373	1.302	18.588

*β*, coefficient of determination; SE, Standard Error; *p*-value, level of significance; OR, odds ratio value; CI, confidence interval.

The X constants shown in this equation are defined in Table 3.

**Table 3:** Definition of the independent variables used in the equation to calculate the risk of malignancy of a thyroid nodule.

Variable	Features
X1	Gender: female = 0; Male = 1
X2	Age: in years
X3	Isthmus location = 1; if left or right lobe = 0
X4	Right lobe location = 1; if isthmus or left lobe = 0
X5	Predominantly or purely cystic content = 1; if predominantly solid or mixed solid-cystic = 0
X6	Predominantly solid content = 1; if predominantly or purely cystic or mixed solid-cystic = 0
X7	Homogeneous echotexture = 1; heterogeneous = 0
X8	Halo: if absent = 1; if present and complete or partially present = 0
X9	Halo: if present and complete = 1; if partially present or absent = 0
X10	Undefined margins = 1; defined = 0
X11	Irregular shape = 1; if regular or lobulated = 0
X12	Lobulated shape = 1; if irregular or regular = 0
X13	Calcification: if absent = 1; if micro or macrocalcifications or peripheral rim calcifications = 0
X14	Calcification: if macrocalcifications = 1; if absent or microcalcifications or peripheral rim calcifications = 0
X15	Calcification: if microcalcifications = 1; if absent or macrocalcifications or peripheral rim calcifications = 0
X16	Absent vascular flow = 1; if predominantly central, peripheral or mixed = 0
X17	Mixed vascular flow = 1; if absent, predominantly central or peripheral = 0
X18	Peripheral vascular flow = 1; if absent, predominantly central or mixed = 0

The application of the prediction formula resulted in a calculated risk of cancer ranging from 0.49% to 97.64% in the present cohort. Dividing this sample according to the calculated risk, it was observed an increasing proportion of cancer cases as the calculated cancer risk rose (Table 4). Twenty cases (7.6%) had a calculated risk ≤3.0%, all of them with proven benign diseases. On the opposite, setting the calculated risk in ≥80%, 21 (8.0%) cases were selected and 85.7% of them confirmed cancer on histopathology. Using these cut-point values, the sensitivity, specificity, accuracy, positive and negative predictive values of the prediction formula were 100%, 86.3%, 92.5%, 85.7%, and 100%, respectively (Table 5).

**Table 4:** Calculated cancer risk applying the statistical tool.

Histopathology	Calculated cancer risk											
	0–10%		10.1–30%		30.1–50%		50.1–70%		70.1–90%		90.1–100%	
	*n*	%	*n*	%	*n*	%	*n*	%	*n*	%	*n*	%
Malignant	6	7.4	15	14.5	6	20.6	8	42.1	11	64.7	11	91.6
Total	81		103		29		19		17		12

**Table 5:** Decision support model.

Cases (%)	7.2	49.8	31.8	3.0	8.0
Calculated cancer risk (%)	0–3	3.1–20	20.1–69.9	70–79.9	80–100
Cancer cases (%)	0	8.4	28.9	50.0	85.7
Clinical management	Observation	Observation/FNAB	FNAB	Surgery/FNAB	Surgery

FNAB, fine-needle aspiration biopsy.

The prediction formula results were stratified according to cytology and the same previous cut-point values were applied. Among the 73 undetermined cases, 10 (13.6%) cases were classified in low risk group and none of them confirmed malignancy. In addition, when the calculated risk was ≥80%, only one case was found, this one with confirmation for carcinoma. Among the 47 cases with non-diagnostic cytology, only 4 cases (8.5%) were set below the inferior cut point (all of them confirmed benignity on histopathology), and only 1 case (2.1%) was set above the cut point (this one confirmed cancer). Among cases with benign, confirmatory and suspicious results for cancer on cytology, the prediction formula was less useful. In the benign group, 4 cases (4.2%) were set above the superior cut-point, only one confirmatory for cancer. On the opposite, among suspicious or confirmatory cases, only one case was set below the inferior cut-point, without confirming cancer on histopathology.

## Discussion

The improved US quality and widespread indication of neck imaging exams resulted in increasing rates of thyroid nodules detection.^1^ According to the American Thyroid Association (ATA) recommendations,^17^ FNAB is the diagnostic method of greater accuracy for detection of cancer among patients with thyroid nodules, while performing cytological examinations in all thyroid nodules is not cost-effective. Some researchers recommend FNAB only in patients with high-risk nodules.^16^, ^18^ The authors found combinations of US characteristics, age and gender information able to accurately predict thyroid cancer. A risk stratification scheme, expressed in relative values (%), allows both patient and surgeon to make a better decision about the recommended treatment. The application of two cut-point values was suggested (≤3.0% and ≥80%), avoiding biopsies in 15.6% of this sample. In fact, FNAB would even increase the number of unnecessary surgeries in the low risk group, as in only 5 (25%) cases cytological results were indicative of benign disease and other 15 (75%) cases would be taken to surgery because of cytological criteria. In the high risk group, FNAB proved to be unnecessary, as cytological results were suggestive or confirmatory for malignancy in 17 (80.9%) cases.

Except for the diameter, all the other variables were included in the statistical formula. Each variable, even with no statistically significant result after multivariate analysis, presents some effect over the result, acting in a dynamic relation net. The authors chose to exclude the diameter from the statistical formula, as it was considered a selection bias. Small nodules submitted to FNAB are usually more suspicious for cancer.

Several studies reported promising results using US to evaluate the risk of malignancy among cases with undetermined,^19^, ^20^ and non-diagnostic cytology.^21^ Despite the low representativeness of both subgroups in the studied sample, it was identified a part of these groups that does not benefit from surgery because of the extremely low risk of cancer, and another part with such a high risk that could be taken to surgical treatment without the need of FNAB. If the proposed inferior cut point were reduced to ≤13%, surgery would be avoided in 21 (44.6%) cases with non-diagnostic cytology, without missing any cancer.

Other authors have already described the US characteristic findings associated to thyroid cancer. The obtained findings were similar to other studies, with some variations when the logistic regression analysis was applied. Koike et al. found irregular shape, solid echo texture, ill-defined margins, hypoechoic characteristics, and fine calcifications as statistically associated with malignancy after multiple regression analysis.^7^ Similar to the author's results, other researchers also found lower mean age as an independent predictor for malignancy after multivariate analysis.^11^, ^22^, ^23^ Gul et al., in a large and well-designed study combining US features together, found margin irregularity, followed by hypoechoic pattern and microcalcifications as the most important US features for malignancy prediction. In their study, the combination of hypoechogenicity, microcalcification, and margin irregularity was found as the most predictive model for cancer (sensitivity of 65.2%, specificity of 98.7%, and PPV of 71.6%).^13^ Some studies compared US characteristics according to mixed benign cytological and malignant histological results.^24^, ^25^, ^26^ Although it was also found association between classical US features and thyroid cancer, this study design can be affected by verification bias, as authors inferred similar accuracy of FNAB and histopathology for thyroid diseases.

Different ways of grouping US characteristics and several kinds of prediction scales were described in literature. Horvath et al. elaborated the Thyroid Imaging Reporting and Data System (TIRADS), taking BI-RADS as a model.^17^ Ito et al. classified US characteristics into 5 levels of risk,^12^ similar to the study of Tomimori et al., which divided US results into four levels.^27^ Kwak et al. noted an increasing risk of malignancy as the number of suspicious US features increased. According to Kwak et al., solid content, hypoechogenicity, microlobulated or irregular shape, presence of microcalcification, and nodules taller than wide were all associated with malignancy after multivariate analysis.^14^ Lin et al. developed a dichotomous US classification: malignant, when solid echo structure, hypoechogenicity, fine calcification, and ill-defined margin were present; and benign, when none of these characteristics were present.^28^

Park et al. used an equation to predict the presence of a malignant nodule, although these authors also included cases with only cytological results in the benign group.^16^ They went further and simplified the malignancy probability for each nodule using a 95% and 99% confidence interval, summarizing the representative US findings in an applicable clinical setting. Nixon et al. produced a nomogram able to predict the need to perform ultrasound-guided FNAB on a thyroid nodule based on biochemical, clinical, and ultrasonography features of 158 patients, all of them submitted to thyroidectomy. Hypoechoic echo texture and microcalcifications had the highest predictive value.^10^

In this present predictive model, the authors standardized the sonographic description before the beginning of the project, using a simple and reproducible methodology, like the one proposed by Andrioli et al.^29^ Some available clinical features (age and gender) were added to a statistical model already explored by other authors,^7^, ^15^, ^16^ bringing this statistical tool to the doctor's office reality. Certainly, this model could be improved including more sonographic and clinical variables, as explored by Nixon et al.,^10^ testing the author's prediction formula in an external sample or confronting their results to other prediction models, like TIRADS.

The analysis of each case by the same radiologist and, in most cases, by the same pathologist, turns it easier to standardize and interpret data, despite increases the risk of bias as there is not a confrontation of this examiner-dependent exam to other opinions. Elaborating a work based on a sample of patients treated in a tertiary referral center might turn it not applicable to a community setting. In part, testing the author's decision support tool in an external sample might bring this prediction model closer to the clinical practice and could minimize both aforementioned biases.

## Conclusions

There was a sufficient basis to observe patients with thyroid nodules under low sonographic risk without using FNAB, even those larger than 1 cm. It was also possible to identify an expressive group at high risk for cancer, dispensing the need of FNAB. The authors’ decision support tool seemed to be practical also in the management of thyroid nodules with undetermined and non-diagnostic cytology. The authors suggested an approach based on an extremely low and an extremely high risk of cancer. Nevertheless, other cases could be included in an observational or more aggressive approach, depending on how many cases each one would be comfortable to miss or to overtreat.

## Conflicts of interest

The authors declare no conflicts of interest.

## References

[1] Davies L., Welch H.G. (2006). Increasing incidence of thyroid cancer in the United States, 1973–2002. JAMA.

[2] Ward E.M., Jemal A., Chen A. (2010). Increasing incidence of thyroid cancer: is diagnostic scrutiny the sole explanation?. Future Oncol.

[3] Kilfoy B.A., Zheng T., Holford T.R., Han X., Ward M.H., Sjodin A. (2009). International patterns and trends in thyroid cancer incidence, 1973–2002. Cancer Causes Control.

[4] Tan G.H., Gharib H. (1997). Thyroid incidentalomas: management approaches to nonpalpable nodules discovered incidentally on thyroid imaging. Ann Intern Med.

[5] Harach H.R., Franssila K.O., Wasenius V.M. (1985). Occult papillary carcinoma of the thyroid: a “normal” finding in Finland. Cancer.

[6] Welker M.J., Orlov D. (2003). Thyroid nodules. Am Fam Phys.

[7] Koike E., Noguchi S., Yamashita H., Murakami T., Ohshima A., Kawamoto H. (2001). Ultrasonographic characteristics of thyroid nodules: prediction of malignancy. Arch Surg.

[8] Camargo R.Y., Tomimori E.K. (2007). Usefulness of ultrasound in the diagnosis and management of well-differentiated thyroid carcinoma. Arq Bras Endocrinol Metab.

[9] Remonti L.R., Kramer C.K., Leitão C.B., Pinto L.C.F., Gross J.L. (2015). Thyroid ultrasound features and risk of carcinoma: a systematic review and meta-analysis of observational studies. Thyroid.

[10] Nixon I.J., Ganly I., Hann L.E., Yu C., Palmer F.L., Whitcher M.M. (2013). Nomogram for selecting thyroid nodules for ultrasound-guided fine-needle aspiration biopsy based on a quantification of risk of malignancy. Head Neck.

[11] Maia F.F., Matos P.S., Silva B.P., Pallone A.T., Pavin E.J., Vassallo J. (2011). Role of ultrasound, clinical and scintigraphic parameters to predict malignancy in thyroid nodule. Head Neck Oncol.

[12] Ito Y., Amino N., Yokozawa T., Ota H., Ohshita M., Murata N. (2007). Ultrasonographic evaluation of thyroid nodules in 900 patients: comparison among ultrasonographic, cytological, and histological findings. Thyroid.

[13] Gul K., Ersoy R., Dirikoc A., Korukluoglu B., Ersoy P.E., Aydin R. (2009). Ultrasonographic evaluation of thyroid nodules: comparison of ultrasonographic, cytological, and histopathological findings. Endocrine.

[14] Kwak J.Y., Han K.H., Yoon J.H., Moon H.J., Son E.J., Park S.H. (2011). Thyroid imaging reporting and data system for us features of nodules: a step in establishing better stratification of cancer risk. Radiology.

[15] Shimura H., Haraguchi K., Hiejima Y., Fukunari N., Fujimoto Y., Katagiri M. (2005). Distinct diagnostic criteria for ultrasonographic examination of papillary thyroid carcinoma: a multicenter study. Thyroid.

[16] Park J.Y., Lee H.J., Jang H.W., Kim H.K., Yi J.H., Lee W. (2009). A proposal for a thyroid imaging reporting and data system for ultrasound features of thyroid carcinoma. Thyroid.

[17] Haugen B.R., Alexander E.K., Bible K.C., Doherty G.M., Mandel S.J., Nikiforov Y.E. (2016). 2015 American Thyroid Association Management Guidelines for adult patients with thyroid nodules and differentiated thyroid cancer: The American Thyroid Association Guidelines Task Force on Thyroid Nodules and Differentiated Thyroid Cancer. Thyroid.

[18] Asanuma K., Kobayashi S., Shingu K., Hama Y., Yokoyama S., Fujimori M. (2001). The rate of tumour growth does not distinguish between malignant and benign thyroid nodules. Eur J Surg.

[19] Yoon J.H., Kwon H.J., Kim E.K., Moon H.J., Kwak J.Y. (2016). The follicular variant of papillary thyroid carcinoma: characteristics of preoperative ultrasonography and cytology. Ultrasonography.

[20] De Napoli L., Bakkar S., Ambrosini C.E., Materazzi G., Proietti A., Macerola E. (2016). Indeterminate single thyroid nodule: synergistic impact of mutational markers and sonographic features in triaging patients to appropriate surgery. Thyroid.

[21] Moon H.J., Kim E.K., Yoon J.H., Kwak J.Y. (2015). Malignancy risk stratification in thyroid nodules with nondiagnostic results at cytologic examination: combination of thyroid imaging reporting and data system and the Bethesda System. Radiology.

[22] Baier N.D., Hahn P.F., Gervais D.A., Samir A., Halpern E.F., Mueller P.R. (2009). Fine-needle aspiration biopsy of thyroid nodules: experience in a cohort of 944 patients. Am J Roentgenol.

[23] Kwong N., Medici M., Angell T.E., Liu X., Marqusee E., Cibas E.S. (2015). the influence of patient age on thyroid nodule formation, multinodularity, and thyroid cancer risk. J Clin Endocrinol Metab.

[24] Butros R., Boyvat F., Ozyer U., Bilezikci B., Arat Z., Aytekin C. (2007). Management of infracentimetric thyroid nodules with respect to ultrasonographic features. Eur Radiol.

[25] Cappelli C., Castellano M., Pirola I., Gandossi E., De Martino E., Cumetti D. (2006). Thyroid nodule shape suggests malignancy. Eur J Endocrinol.

[26] Iannuccilli J.D., Cronan J.J., Monchik J.M. (2004). Risk for malignancy of thyroid nodules as assessed by sonographic criteria: the need for biopsy. J Ultrasound Med.

[27] Tomimori E.K., Bisi H., Medeiros-Neto G., Camargo R.Y. (2004). Ultrasonographic evaluation of thyroid nodules: comparison with cytologic and histologic diagnosis. Arq Bras Endocrinol Metabol.

[28] Lin J.H., Chiang F.Y., Lee K.W., Ho K.Y., Kuo W.R. (2009). The role of neck ultrasonography in thyroid cancer. Am J Otolaryngol.

[29] Andrioli M., Carzaniga C., Persani L. (2013). standardized ultrasound report for thyroid nodules: the endocrinologist's viewpoint. Eur Thyroid J.

